# Health preparedness plan for dengue detection during the 2020 summer Olympic and Paralympic games in Tokyo

**DOI:** 10.1371/journal.pntd.0006755

**Published:** 2018-09-20

**Authors:** Naoki Yanagisawa, Koji Wada, John D. Spengler, Ramon Sanchez-Pina

**Affiliations:** 1 Department of Environmental Health, Harvard T. H. Chan School of Public Health, Boston, Massachusetts, United States of America; 2 Graduate School of Public Health, International University of Health and Welfare, Tokyo, Japan; University of Tokyo, JAPAN

## Abstract

**Background:**

Participants in mass gathering events are at risk of acquiring imported and locally endemic infectious diseases. The 2014 dengue outbreak in Tokyo gathered attention since it was the first time in 70 years for Japan to experience an autochthonous transmission. Preparation for emerging infectious threats is essential even in places where these outbreaks have been largely unknown. The aim of this study is to identify strategies for early detection and prevention of dengue infection during the 2020 summer Olympics and Paralympics in Tokyo.

**Methodology/Principal findings:**

We modified and adapted the failure mode and effect analysis (FMEA) methodology, generally used in industrial manufacturing, to examine the current controls for dengue detection and assessment. Information on existing controls were obtained from publicly available resources. Our analysis revealed that the national infectious disease control system to detect dengue in Japan is robust. However, in the case of large assemblies of international visitors for special events when the spread of communicable and vector-borne diseases increases, there are three main gaps that could be reinforced. First, cyclical training or a certification program on tropical disease management is warranted for physicians, especially those working in non-infectious disease-designated hospitals or clinics. Second, multi-language communication methods need to be strengthened especially in the health and hospitality sector. Third, owners of accommodations should consider incorporating a formal tropical disease-training program for their staff members and have a contingency plan for infectious disease-suspected travelers.

**Conclusions/Significance:**

Our findings may facilitate physicians and public health officials where new controls would be beneficial for the 2020 summer Olympics and Paralympics. The FMEA framework has the potential to be applied to other infectious diseases, not just dengue.

## Author summary

Dengue is a mosquito-borne disease that is most prevalent among the emerging arboviruses. Most patients recover from dengue without complications, but a small portion of cases may progress to severe dengue which carries a high mortality rate if left untreated. In 2014, a dengue outbreak unexpectedly occurred in Tokyo, which was the first time in 70 years for Japan to experience an autochthonous transmission. Thus, preparation for dengue and other emerging infectious threats is essential even in places where these outbreaks have been largely unknown. Tokyo will be hosting the Olympic and Paralympic games in 2020, and interventions are warranted to mitigate the risks. We modified and adapted the failure mode effect analysis (FMEA) methodology to test the vulnerability and resiliency of the current controls. Although the FMEA methodology is generally used in industrial manufacturing, it has the potential to be utilized for health preparedness for other infectious diseases as well. Our analysis identifies three strategies to reinforce early detection of dengue infection and prevent further transmission during the Olympic and Paralympic Games.

## Introduction

Participants and spectators of international sporting events such as the Olympics, are at risk of acquiring imported and locally endemic infectious diseases [1, 2]. Host countries need to be well prepared to address these challenges, since infectious disease outbreaks can occur with little warning. The risk of an outbreak occurring, even in areas where a disease has been previously unknown, is increasing due to globalization. The International Air Travel Association reported that there were 3.8 billion air travelers in 2016 but expects the number to nearly double to 7.2 billion passengers in 2035 [3]. Globalization has certainly provided a great opportunity for traveling, culture exchange, and trade but on the other hand, it has enabled a person carrying an infectious pathogen to go almost anywhere in the world within days. Development of the transportation network expedites the rate at which a pathogen spreads and poses a major threat to the international community [4]. It is notable, however, that despite the ever-increasing and unpredictable shocks of health pandemics, the tourism industry continues to grow, accounting for 10.2% of the global gross domestic product in 2016 [5].

Respiratory infections and gastrointestinal illnesses have always been a major concern at international sporting mass gatherings [6]. Examples of these outbreaks include influenza at the 2002 Winter Olympics in Salt Lake City [7], norovirus gastroenteritis at the 2006 FIFA World Cup in Germany [8], and measles at the 2010 Winter Olympics in Vancouver [9]. Recently, a norovirus outbreak was confirmed at the 2018 Winter Olympics in PyeongChang [10]. However, vector-borne disease, namely Zika virus, gathered worldwide attention at the 2016 Olympics in Rio de Janeiro [11, 12]. The outbreak raised considerable debate internationally about whether postponing and/or relocating the games should be considered, due to the risk of infection of athletes and visitors and potential acceleration of the spread of Zika virus worldwide [13–16]. The World Health Organization declared that there was no public health justification for postponing or cancelling the 2016 Olympic Games, and no laboratory-confirmed cases of Zika virus were subsequently reported among participants [17, 18].

### The 2020 summer Olympic and Paralympic games in Tokyo

Tokyo will be hosting the next Olympic and Paralympic games during the summer season in 2020 (Tokyo 2020) [19]. Although Tokyo is located in a temperate climate zone, vector-borne disease will continue to be a major concern, as it was with the previous games. It was unexpected that a dengue outbreak occurred in the summer of 2014, which was the first time in 70 years for Japan to experience an autochthonous transmission [20]. This outbreak implies that Tokyo possess a suitable ecoclimatic condition, has a population that is immunologically naïve to the virus, with a high probability of introduction of the virus by travelers, and the existence of the *Aedes* mosquito, which is the competent vector. Furthermore, the activity of the *Aedes* mosquito and the number of imported dengue cases will peak during the same time period as Tokyo 2020 [21]. Although *A*. *aegypti* does not exist in Japan, *A*. *albopictus* is found in Tokyo and seems to be extending its habitat further north, where climate change may play an important role [22].

Prior to this dengue outbreak, which resulted in more than 160 laboratory-confirmed cases, there have been 50 to 200 official reported dengue cases annually, all of which have been imported [21]. Similar to the global trend, the number of tourists who visited Japan increased 4.6-fold, from 5.2 to 24.0 million people [23], from 2003 to 2016. Many factors contribute to risk of dengue importation, such as number and origin of the tourists, epidemiology of dengue in their countries of origin, and seasonal synchrony. In this regard, Japan may be at increased risk for dengue importation since the country is promoting more tourists, aiming for 40 million people by 2020, from all over the world.

### Health effects of dengue

Dengue is the most prevalent among the emerging arboviruses. Recent studies have demonstrated that 58.4 to 96 million clinically significant cases occur annually, with the number of cases more than doubling every decade between 1990 and 2013 [24, 25]. Clinical characteristics of dengue include sudden high-grade fever, headache, vomiting, myalgia and arthralgia, typically occurring in what is called the febrile stage. These symptoms typically develop between 4 and 7 days after the bite of an infected mosquito. This phase lasts for 3 to 7 days, after which most patients recover without complications. However, among the infected, up to 5% may progress to severe dengue, an illness characterized by plasma leakage leading to hypovolemic shock, hemorrhage, and potentially death [26]. The mortality rate of severe dengue is 20% if left untreated, but it could be reduced to less than 1% with appropriate clinical management [27]. While dengue could result in severe consequences, it is notable that most dengue infections are asymptomatic or mild—only about 1 in 4 infections is symptomatic—and result in complete recovery [28]. However, people with asymptomatic dengue infections may be more infectious to mosquitoes than people with dengue symptoms [29]. This implies that an asymptomatic infected individual can be the source of virus for mosquitos as well.

### Health preparedness plan and failure mode and effects analysis (FMEA)

Preparation for emerging infectious threats is essential even in places like Japan where these outbreaks have been largely unknown. Considering the nature of infectious disease, it is virtually impossible to prevent any pathogen from entering a country just by enhancing border control. A pragmatic approach is to have a preparedness plan so that health professionals along with others knows how to recognize symptoms and how to respond. In this context, failure mode and effects analysis (FMEA) is a framework that is suitable to test the vulnerability and resiliency of the current preparedness plans and to strengthen these current plans in order to forestall failures [30].

FMEA is a procedure for the analysis of potential failure modes within a system in order to classify and quantify risks by their occurrence, severity and detection controls already in place. The original FMEA methodology was developed by the U.S. Military during World War II for the assessment of failures and subsequent risks [31]. Although the process and the parameters continue to evolve to conform with new applications, the FMEA basic framework is retained and used around the world 70 years after the end of World War II. After the U.S. Military developed FMEA, it was adopted by the National Aeronautics and Space Agency, and then by various large U.S. manufacturing companies. General Motors and Boeing Aircraft use FMEA methodology to assess their manufacturing processes. When some of the models of Toyota Motors experienced a sudden unintended acceleration and catastrophic accidents, the company incorporated FMEA in a significant way to assess the risks and prevent accidents.

FMEA became well-known because it is a risk prevention tool instead of a corrective measurement to diminish risk when designing new products, industrial processes, customer service applications, and infrastructure of the environment and health systems. The methodology of FMEA is appealing, because the analysis is prospective rather than retrospective, enabling us to not rely on the analysis of errors after they have occurred. FMEA has been implemented mainly in the engineering industry and is more recently gaining momentum in healthcare [32–34]. However, to the best of our knowledge, the FMEA framework has not been applied to health preparedness plans for infectious diseases. The aim of this study is to search the gaps in the current controls which could be reinforced for dengue detection and assessment during Tokyo 2020 using the FMEA methodology.

## Methods

### Application of FMEA

In the application of FMEA, a process map of the operation is created, including all of the activities and understanding how the process steps are related. Second, every operation or activity in the process will be analyzed in order to understand its potential failure modes and the effects of these failures. Third, the controls for each failure mode will are described, and the severity (S), occurrence (O) and detection (D) of each failure mode under current process controls is assessed. We have developed a novel criterion for each of the components (**S1 Table, S2 Table, S3 Table**). The numbers are multiplied to calculate the Risk Priority Number (RPN) for every failure mode (RPN = S x O x D; range, 1 to 1000). The criteria to take action varies, but the most accepted notions to take action after the RPNs are calculated are as follows: 1. Create a corrective action for every failure mode that has a 9 or a 10 in any of its S, O, or D numbers or RPN number higher than 150; 2. Decide if a corrective action is necessary for failure modes with RPNs between 80 and 150. Implement a corrective action if the cost of the corrective control is low or moderate. 3. Apply a corrective control when the RPN is lower than 80 if the corrective action is easy, inexpensive and quick to implement. 4. The “safe” range is for failure modes with RPNs within the range of 1 to 40. Lastly, after the corrective actions have been implemented in the design/process, the new RPN should be checked to confirm the improvements (risk reduction of failure mode). A countries’ health systems or infrastructure and the situation of the disease in which may occur should be taken into consideration upon evaluation. The most relevant number should be chosen if there is no exact match.

In our analysis, we focused specifically on processes necessary for detection and assessment of dengue cases. Since Japan is currently not endemic for dengue, physicians may not be prepared or even aware that a dengue patient could show up in their own hospital. In order to promptly activate a public health response, such as enhancing the surveillance system or vector control activities in areas where persons became infected, accurate and timely reporting of the disease will be essential. Therefore, we divided the process into three components: 1. detection of the disease, 2. assessment of the disease, 3. patient communication. Detection of the diseases focuses mainly on the role the travelers themselves and the community, whereas assessment of the disease focuses on the role of the physicians in the hospitals and/or clinics. For the purpose of our study, we did not include other interventions for prevention or mitigating disease spread for analysis such as vector control strategies, disease/vector surveillance, and community education. The authors of the manuscript are experts in the field of clinical infectious diseases, public health, disaster preparedness, and environmental health.

### Data collection

All data were obtained from publicly available resources. The majority of the data regarding the current controls came from the websites of the Japan National Tourism Organization (JNTO), The Ministry of Health, Labour and Welfare (MHLW) of Japan, the National Institute of Infectious Diseases (NIID) of Japan, and The Japanese Association for Infectious Diseases (JAID). Collecting ground-level firsthand information was not conducted, although there were a few direct conversations with individuals of their personal experiences.

## Results

We identified a total of 20 failure modes which are summarized in **Tables 1–3**. There were 8 failure modes for detection of disease, 6 for assessment of disease, and 6 for patient communication. Failure modes related to Olympic-related facilities (No. 6 and No. 20) were not evaluated since there was no publicly available information for current controls.

**Table 1:** Characteristics of failure and risk priority number (Process: Detection of disease).

No	Failure mode	Causes of Failure	Current Controls	Effect of Failure of process/system	S	O	D	RPN
1	Travelers enters a country despite symptoms such as fever.	Lack of equipment and/or education for airport staff members to detect dengue.	Routine thermographic inspection before passport check-in desk. Procedure manual for airport staffs to follow.	Increase the probability of spreading dengue in the community during the summer season. Patients’ delay in seeking diagnosis/treatment.	6	2	3	36
2	Travelers do not visit airport clinics despite symptoms or medical concerns	Lack of awareness about infectious diseases or reluctant to visit the clinic for personal reasons.	Digital displays/posters reminding and encouraging travelers to visit the airport clinic if feeling sick or had any exposure to animals/birds/mosquitoes.	Increase the probability of spreading dengue in the community during the summer season. Patients’ delay in seeking diagnosis/treatment.	6	3	5	90
3	Missed cases at public places (transportation hubs, landmark tourist places, etc.)	Staff at public places are unable to properly give assistance when travelers seek help	Major tourist sites and railway stations are equipped with free maps and multilingual leaflets. Quality of assistance varies and relies on individual ability.	Increase the probability of spreading dengue in the community during the summer season. Patients’ delay in seeking diagnosis/treatment.	6	2	7	84
4	Missed cases at hotels	Hotel staff members are unable to give assistance when travelers seek help	There is a front desk in case of any need of assistance. Majority of hotels have information booklet guiding travelers when feeling sick.	Increase the probability of spreading dengue in the community during the summer season. Patients’ delay in seeking diagnosis/treatment.	6	4	3	72
5	Missed cases at vacation rentals	Owners/staffs are unable to properly give assistance when travelers seek help	No requirements for having a front desk. Availability of assistance varies significantly.	Increase the probability of spreading dengue in the community during the summer season. Patients’ delay in seeking diagnosis/treatment.	6	6	8	288
6	Missed cases at Olympic-related facilities	Lack of training about how to identify dengue among workers/volunteers	No publicly available information	Increase the probability of spreading dengue in the community during the summer season. Patients’ delay in seeking diagnosis/treatment.	Not evaluated yet			
7	Missed cases when traveling with one’s sport team	Team coaches/staff members lack knowledge about dengue/tropical disease.	Each country has their medical team that they could receive advice from.	Increase the probability of spreading dengue in the community during the summer season. Patients’ delay in seeking diagnosis/treatment.	6	4	3	72
8	Travelers do not go to hospitals/clinics to get medical treatment despite symptoms	Insufficient information about nearby hospital/clinics or reluctant to go because of health insurance issues, etc.	Website listing medical institutions that provide treatment in English/Chinese/Korean. Telephone service providing information about medical institutions and health insurance system in English/Chinese/Korean/Thai/Spanish.	Increase the probability of spreading dengue in the community during the summer season. Patients’ delay in seeking diagnosis/treatment.	6	5	4	120

Abbreviations: S, severity; O, occurrence; D, detection; RPN, risk priority number.

**Table 2:** Characteristics of failure and risk priority number (Process: Assessment of disease).

No	Failure mode	Causes of Failure	Current Controls	Effect of Failure of process/system	S	O	D	RPN
9	Physicians fails to diagnose cases at airport clinics	Lack knowledge/medical equipment to identify travelers with dengue-related symptoms	Physicians with knowledge on tropical diseases at airport clinics. Guidance to visit infectious disease-designated hospitals when suspected.	Doctors' delay in the diagnosis and/or treatment may endanger human life. Delay in the surveillance system to detect a possible dengue outbreak.	6	4	4	96
10	Physicians fail to diagnose cases at infectious disease designated hospitals	Lack knowledge/medical equipment to diagnose dengue/ tropical diseases	Majority of hospitals have infectious disease board-certified physicians in service. Rapid antigen tests available in most places. Number of imported dengue cases updated every month.	Doctors' delay in the diagnosis and/or treatment may endanger human life. Delay in the surveillance system to detect a possible dengue outbreak.	6	3	2	36
11	Physicians fail to diagnose cases at non-infectious disease-designated hospitals or clinics	Lack knowledge/medical equipment to diagnose dengue and/or tropical diseases	Publicly available guideline on mosquito-borne infections. Rapid antigen tests not available in most places. No cyclical training/certification program to identify dengue/other tropical diseases among the medical community.	Doctors' delay in the diagnosis and/or treatment may endanger human life. Delay in the surveillance system to detect a possible dengue outbreak.	6	6	7	252
12	Physicians fail to diagnose cases at hospitals/clinics at night	Lack of knowledge among emergency physicians. Infectious disease physicians may not be available or on-call.	Publicly available guideline on mosquito-borne infections. Detection heavily relies on the emergency physician’s individual skills.	Doctors' delay in the diagnosis and/or treatment may endanger human life. Delay in the surveillance system to detect a possible dengue outbreak.	6	5	7	210
13	Physicians fail to refer/transfer patients to a hospital with experience in dengue	Insufficient information on which hospitals to refer/transfer patients	There is a formal registry of hospitals that can treat dengue and other mosquito-borne infections. The information is available in Japanese.	Doctors' delay in the diagnosis and/or treatment may endanger human life. Delay in the surveillance system to detect a possible dengue outbreak.	6	2	3	36
14	Physicians fail to report cases to the public health department	Physicians do not know that dengue needs to be reported or just forget to report	Medical school education teaches dengue cases are required to be reported immediately after diagnosis by law. There is no penalty for not reporting.	Delay in the surveillance system to detect a possible dengue outbreak.	6	2	4	48

Abbreviations: S, severity; O, occurrence; D, detection; RPN, risk priority number.

**Table 3:** Characteristics of failure and risk priority number (Process: Patient communication).

No	Failure mode	Causes of Failure	Current Controls	Effect of Failure of process/system	S	O	D	RPN
15	Communication failure at hospitals/clinics	Lack of personnel or service to help communicate with medical staffs	Few hospitals have multi-language translators available or on-call. There is an accreditation system to ensure sufficient communication with foreign travelers.	Unable to acquire accurate information needed for diagnosis and/or treatment.	6	7	4	168
16	Communication failure at hospitals/clinics	Patients unwilling to give proper information to physicians	Few hospitals have protocols to check if patients are unresponsive due to drug problems, culture, or fear of cost.	Unable to acquire accurate information needed for diagnosis and/or treatment.	6	2	6	72
17	Communication failure at public places (transportation hubs, landmark tourist places, etc.)	Lack of personnel who could communicate in foreign languages	Major public places equip multilingual leaflets. In-person communication skills relies on individual ability. Several tourist information centers have full-time service available in English and other 2 languages.	Unable to acquire accurate information needed for prompt referral/transferal to medical institutions.	6	4	4	96
18	Communication failure at hotels	Lack of personnel who could communicate in foreign languages	Majority of hotels have staffs who could communicate in English. Multilingual assistance may be difficult.	Unable to acquire accurate information needed for prompt referral/transferal to medical institutions.	6	4	4	96
19	Communication failure at vacation rentals	Lack of personnel who could communicate in foreign languages.	No formal procedure to have translators available or on-call to listen and interpret patients' symptoms properly. Communication in foreign languages is not mandatory.	Unable to acquire accurate information needed for prompt referral/transferal to medical institutions.	6	7	8	336
20	Communication failure at Olympic-related facilities	Lack of personnel or service to help patients communicate in foreign languages	No publicly available information	Unable to acquire accurate information needed for prompt referral/transferal to medical institutions.	Not evaluated yet			

Abbreviations: S, severity; O, occurrence; D, detection; RPN, risk priority number.

Overall, the current controls for dengue detection in Japan is robust. There was no failure mode that had 9 or a 10 in any of its S, O, or D numbers, and 13 with RPN number less than 150. There is a comprehensive guideline on mosquito-borne infection and a list of medical institutions capable of taking care of dengue-suspected travelers, both of which are written in Japanese. The number of imported dengue cases in Japan is updated every month and is publicly available, enabling physicians to be up-to-date on the current epidemiological situation. There are websites and telephone services in multi-languages to facilitate travelers when assistance in needed. All of these current controls may enable travelers with symptoms to be detected and transferred to the proper medical institutions for assessment and treatment. We could enhance the current controls for detection and prevention through further educating the medical community and establish a sense of share responsibility among the hospitality sector and well as within the event organizers.

There were 5 failure modes that had RPNs higher than 150. In the case of large assemblies of international visitors for special events when the spread of communicable and vector-borne diseases increases, these gaps needs to be especially taken in consideration for reinforcement. The failure modes were as follows: 1. missed cases at vacation rentals (No. 5, RPN 288); 2. physicians fail to diagnose cases at non-infectious disease designated hospitals or clinics (No. 11, RPN 252); 3. physicians fail to diagnose cases at hospitals/clinics at night (No. 12, RPN 210); 4, communication failure at hospitals/clinics (No. 15, RPN 168), 5. communication failure at vacation rentals (No. 19, RPN 336). **Tables 4–6** summarizes the recommendations/action plans and the RPNs for the failure modes after corrective actions have been implemented. Owners of vacation rentals may implement formal training seminars about dengue and other tropical infectious diseases for their staff members. There should be contingency plans for infectious disease-suspected travelers. Capacity building on tropical diseases, especially for physicians working in non-infectious disease-designated hospitals and emergency physicians, may be important. Communication methods could be improved in every sector by incorporating a translator or a medical telephone service not just in the English language.

**Table 4:** Recommendations and ratings (Process: Detection of disease).

No	Recommendations	Action Plan	S	O	D	RPN
1	Enhance current controls	Provide opportunities to update knowledge on tropical diseases. In order to detect the disease and report them to health authorities effectively, staff members need constant training.	6	2	2	24
2	Raise awareness of the potential infection they might have acquired while abroad	Provide infectious disease information on airplane monitors in various languages. Encouragement by airport staff members to visit airport clinics when travelers seem to be feeling sick.	6	3	3	54
3	Provide capacity building assistance to the tourism/transportation business	Inform tourism/transportation stakeholders on how to prevent infection. Implement a training seminar on dengue/other tropical diseases. Develop protocols on how to guide travelers to proper medical institutions.	6	2	4	56
4	Provide capacity building assistance to the tourism/hospitality business	Inform tourism/hospitality stakeholders on how to prevent infection. Implement a training seminar on dengue/other tropical diseases. Establish contingency plans for infectious disease-suspected travelers.	6	4	2	56
5	Provide capacity building assistance to the tourism/hospitality business	Inform tourism/hospitality stakeholders including vacation rental owners on how to prevent infection. Implement a training seminar on dengue/other tropical diseases. Establish contingency plans for infectious disease-suspected travelers.	6	6	4	144
6	Not evaluated yet					
7	Enhance current controls	Make sure that sports team coaches and accompanying staff be made aware of not just dengue but other communicable and vector borne diseases that have or could occur at mass gatherings.	6	4	2	48
8	Enhance current controls	Familiarize the website and telephone service to wide range of tourists. The tourism/hospitality business could give assistance. Increase the number of languages that could be supported.	6	5	3	90

Abbreviations: S, severity; O, occurrence; D, detection; RPN, risk priority number.

**Table 5:** Recommendations and ratings (Process: Assessment of disease).

No	Recommendations	Action Plan	S	O	D	RPN
9	Enhance current controls	Check routinely about the coordination system of related organization in executing a response to infections. Continue educating personnel on how to react to hypothetical situations, such as if individual exhibits symptoms of infection. Equip latest rapid detection assays for dengue/other tropical diseases.	6	2	2	24
10	Enhance current controls	Check routinely about the coordination system of related organization in executing a response to infections. Continue educating personnel on how to react to hypothetical situations, such as if individual exhibits symptoms of infection. Equip latest rapid detection assays for dengue/other tropical diseases.	6	2	2	24
11	Provide capacity building assistance to health care workers	Implement cyclical training or a certification program for dengue/other tropical diseases among the medical community. Build strategies for prompt referral/transferal of patients when assistance is needed.	6	4	4	96
12	Provide capacity building assistance to health care workers	Provide educational program for dengue/other tropical diseases by infectious disease specialists. Build strategies for prompt referral/transferal of patients when assistance is needed.	6	4	4	96
13	Routine communication between hospitals	Familiarize the website listing the formal registry of hospitals that could handle mosquito-borne disease. Build strategies for prompt referral/transferal of patients when assistance is needed.	6	1	3	18
14	Enhance communication among physicians and laboratory technicians	Develop an accountability checking system to ensure that the information is reported to the public health department	6	2	2	24

Abbreviations: S, severity; O, occurrence; D, detection; RPN, risk priority number.

**Table 6:** Recommendations and ratings (Process: Patient communication).

No	Recommendations	Action Plan	S	O	D	RPN
15	Ensure hospitals/clinics have translators available or on-call in various languages	Promote and incentivize hospital/clinics to get accreditation for servicing international travelers. Encourage institutions to implement a telephone medical services if hiring translators is not feasible.	6	2	4	48
16	Enhancement of contingency protocols	Develop a protocol to deal with unresponsive patients	6	1	6	36
17	Enhance current controls	Continue effort to increase the number of places where travelers could receive multilingual assistance.	6	2	4	48
18	Enhance current controls	Continue effort to increase the number of staff members who could communicate in foreign languages.	6	2	4	48
19	Ensure vacation rentals to have translators available or on-call in multi-languages	Inform tourism/hospitality stakeholders about the need for translators or services to facilitate traveler’s needs.	6	4	4	96
20	Not evaluated yet					

Abbreviations: S, severity; O, occurrence; D, detection; RPN, risk priority number.

## Discussion

To the best of our knowledge, this is the first study to utilize FMEA analysis to identify opportunities for improving detection and mitigation of a particular infectious disease. Application of FMEA of public health protection in associating with large international event is also novel. FMEA analysis identified ways the current devised controls for infectious disease could be improved in this context. First, cyclical training or a certification program on tropical disease management is warranted for physicians, especially those working in non-infectious disease-designated hospitals or clinics. Second, multi-language communication methods must be strengthened especially in the health and hospitality sector. Third, owners of accommodations should consider incorporating a formal tropical disease-training program for their staff members and have a contingency plan for infectious disease-suspected travelers.

Travelers may have lower chances of being diagnosed correctly when they visit non-infectious disease-designated hospitals or clinics. As of January 2017, there are 649 hospitals and 13,185 clinics in Tokyo, which accounts for 7.7% and 13.0% of all of the hospitals and clinics countrywide, respectively [35]. However, there are only 1,049 board-certified infectious disease physicians working in medical institutions in Japan as of December 2014 [36]. Moreover, there are only 492 physicians working in infectious disease departments, which is 0.2% of the total working physicians in hospitals and clinics [37]. The scarcity of infectious disease specialists in Japan was pointed out during the time when the Ebola outbreak occurred in West Africa as well [38]. In addition, many physicians do not have experience in dengue management since it is not a common disease in Japan as of date. Considering that the Japanese healthcare system enables free-access to any hospital or clinic, chances may be quite high for travelers with suspected dengue to be seen first by a physician with little or no experience in tropical infectious disease. After the dengue outbreak in 2014, the MHLW and NIID issued a comprehensive, publicly available guideline written in Japanese for managing mosquito-borne infections [39]. The NIID releases epidemiological data on the trends of imported dengue cases every month [40]. JAID provides list of medical institutions that are capable and have experience in treating mosquito-borne diseases [41]. All of these are useful tools, but in order to fully utilize them, physicians, especially those working in non-infectious disease-designated hospitals, first need to be aware of the disease and understand that they might be the ones who will see the patients first-hand. To that end, implementing cyclical training or a certification program for dengue and other tropical diseases among the medical community may be beneficial.

Communication difficulty with travelers is a common challenge that every sector faces. It is especially important, however, for medical institutions to be prepared, since obtaining accurate information from patients is essential to making the correct diagnosis. Traditionally, medical institutions relied heavily on a physician’s personal ability to communicate in English. In addition, to arrange for a translator, especially in a non-English language, physicians had to rely mainly on their personal networks. To establish a system to support non-Japanese residents in Japan and visitors from other countries, an accreditation system (Japan Medical Services Accreditation for International Patients: JMIP) was implemented and established by the MHLW in fiscal year 2011. This was done as a part of national project which promotes the smooth acceptance of international patients in Japanese medical settings. As of February 2018, there are 36 accredited medical institutions in Japan, with 9 in Tokyo [42]. In addition, there are private sectors that offer healthcare support for internationals in Japan. For example, a medical translation service to facilitate hospitals operated by the Japanese Institute of Global Health is available in 17 languages as of February 2018 [43]. For the tourists in Tokyo, there is a telephone information service provided in English, Chinese, Korean, Thai, and Spanish that takes questions daily about medical institutions and the health insurance system in Japan [44]. JNTO has a website that lists medical institutions that can provide treatment to international visitors in English, Chinese, and Korean [45]. Many efforts are being implemented at a rapid pace, but there are still areas that need to be improved. The MHLW has a website that provides information on hospitals/clinics and the links to each prefectures’ websites, but states that almost all of the information on these websites is offered only in Japanese [46].

Outside of the medical setting, the tourism and hospitality sector need to know how to respond when travelers seek assistance. This may not be a huge concern for major hotels since there is often front desk open 24 hours a day with staff members capable of speaking foreign languages to provide any assistance when needed. However, this may be problematic for vacation rentals, which are rapidly growing in usage and popularity. To start a vacation rental service in Japan, owners or managers need to adhere to the Inns and Hotels Act as a general rule [47]. As of March 2015, there are 9,879 hotel businesses (hotel-gyo), 41,899 ryokan businesses (ryokan-gyo), and 26,349 budget hotel businesses (kan-i-syukuhaku-gyo), which is classified by the Act [48]. It is typical to obtain a budget hotel business operating license to start a vacation rental. Although local governments have their own policies, the Act itself does not require budget hotel businesses to have a front desk, whereas the other two styles are required to do so [47]. There are no regulations for placing a translator or any other means of communication in foreign languages as well. Both may hinder travelers from getting the proper assistance when needed most. In addition, unauthorized operation of vacation rental has been reported [49]. To ensure the proper operation of vacation rentals and fulfill the increased demand for accommodations by tourists, a new law, the Private Lodging Business Act, will be in effect starting in June 2018 [50, 51]. This Act states that owners need to ensure the comfort and convenience of foreign tourists, meaning that communications tools in foreign languages are needed which is a step forward from the previous Act. In addition, owners need to ensure the hygiene and safety of the guests. Thus, a tropical disease-training seminar to staff members and having a contingency plan for infectious disease-suspected travelers may be beneficial.

We specifically applied the FMEA framework to health preparedness for dengue infection for Tokyo 2020. However, this framework could be expanded and tailored to other diseases or mass gatherings as well. Given that dengue was introduced, chikungunya and Zika could be problematic as well. Although an outbreak has not been recognized in Japan to date, there is always the possibility that these infections would cause an outbreak. The previous chikungunya outbreak in Italy, where *A*. *albopictus* played a major role, implies that an urban cycle could be formed in a non-tropical area [52]. Zika outbreak would be less of a possibility since *A*. *albopictus* has a lower vector competence than *A*. *aegypti* [53, 54], but there is always the possibility for a virus to evolve and attain competence. The framework could be also adapted to more lethal infections such as Ebola. When performing a FMEA analysis, expertise with specific knowledge about the various processes are needed to estimate the severity, occurrence and detection numbers in order to have an objective assessment of the inherent risk of every potential failure mode.

There are several limitations that need to be addressed. First, we based our evaluation on publicly available information. Therefore, our evaluation may not be perfectly accurate or up-to-date. Key stakeholders may claim that there are already controls implemented and therefore our scoring might not be accurate. However, the main goal of our proposal was to demonstrate a framework that may be useful in preparedness planning to fill in the gaps as much as possible to mitigate health risks. In addition, we could not evaluate failure modes on Olympic-related facilities such as stadiums, Olympic villages, or any transportation offered by the Olympic Committee, since we could not find publicly available information. Second, our proposed approach may not have clear benefit in terms of reducing the morbidity and mortality of dengue in Tokyo 2020. However, the purpose of our study is to find the gaps that may hinder detection and prompt referral/transferal of travelers suspected of dengue. Rapid and accurate disease detection and assessment is one of the essential aspects of preventing disease to spread. Considering the fact that asymptomatic patients may be more infectious to mosquitoes than people with dengue symptoms, a multidisciplinary approach such as vector control, target surveillance, environmental intervention, and community education are needed to ultimately meet the goals.

Although FMEA analysis described has been applied to health-preparedness plan for the potential outbreak of dengue at the 2020 summer Olympic and Paralympic Games in Tokyo, it certainly could be extended to other infectious diseases. We focused on dengue because of a prior outbreak in Japan and the fact that dengue mortality is increasing. The trend is expected to continue due to factors such as urbanization and climate variability [55].

## Supporting information

S1 Table Criteria for the evaluation of severity component. (DOCX)

S2 Table Criteria for the determination of occurrence component. (DOCX)

S3 Table Criteria for the determination of detection component. (DOCX)

## References

[1] McCloskey B, Endericks T, Catchpole M, Zambon M, McLauchlin J, Shetty N, et al London 2012 Olympic and Paralympic Games: public health surveillance and epidemiology. Lancet. 2014;383:2083–9. 10.1016/S0140-6736(13)62342-9 24857700 PMC7138022

[2] Smallwood CAH, Arbuthnott KG, Banczak-Mysiak B, Borodina M, Coutinho AP, Payne-Hallstrom L, et al Euro 2012 European Football Championship Finals: planning for a health legacy. Lancet. 2014;383:2090–7. 10.1016/S0140-6736(13)62384-3 24857705

[3] International Air Transport Association (IATA). IATA Forcasts Passenger Demand to Double Over 20 Years. [cited 20 March 2018]. Available from: http://www.iata.org/pressroom/pr/Pages/2016-10-18-02.aspx

[4] Tatem AJ, Jia P, Ordanovich D, Falkner M, Huang Z, Howes R, et al The geography of imported malaria to non-endemic countries: a meta-analysis of nationally reported statistics. Lancet Infect Dis. 2017;17:98–107. 10.1016/S1473-3099(16)30326-7 27777030 PMC5392593

[5] World Travel and Tourism Council (WTTC). Travel and Tourism, Global Economic Impact and Issues 2017. [cited 20 March 2018]. Available from: https://www.wtttc.org/-/media/files/reports/economic-impact-research/2017-documents/global-economic-impact-and-issues-2017.pdf

[6] Abubakar I, Gautret P, Brunette GW, Blumberg L, Johnson D, Poumerol G, et al Global perspectives for prevention of infectious diseases associated with mass gatherings. Lancet Infect Dis. 2012;12:66–74. 10.1016/S1473-3099(11)70246-8 22192131

[7] Gundlapalli AV, Rubin MA, Samore MH, Lopansri B, Lahey T, McGuire HL, et al Influenza, Winter Olympiad, 2002. Emerg Infect Dis. 2006;12:144–6. 10.3201/eid1201.050645 16494733 PMC3291389

[8] Schenkel K, Williams C, Eckmanns T, Poggensee G, Benzler J, Josephsen J, et al Enhanced surveillance of infectious diseases: the 2006 FIFA World Cup experience, Germany. Euro Surveill. 2006;11:234–8. 17370965

[9] Memish ZA, Stephens GM, Steffen R, Ahmed QA. Emergence of medicine for mass gatherings: lessons from the Hajj. Lancet Infect Dis. 2012;12:56–65. 10.1016/S1473-3099(11)70337-1 22192130 PMC7185826

[10] ProMED Mail. Norovirus-South Korea:(Gangwon) Winter Olympics. [cited 19 February, 2018]. Available at: www.promedmail.org/direct.php?id=20180209.5616795

[11] Elachola H, Gozzer E, Zhuo J, Memish ZA. A crucial time for public health preparedness: Zika virus and the 2016 Olympics, Umrah, and Hajj. Lancet. 2016;387:630–2. 10.1016/S0140-6736(16)00274-9 26864962 PMC7138056

[12] Petersen E, Wilson ME, Touch S, McCloskey B, Mwaba P, Bates M, et al Rapid Spread of Zika Virus in The Americas—Implications for Public Health Preparedness for Mass Gatherings at the 2016 Brazil Olympic Games. Int J Infect Dis. 2016;44:11–5. 10.1016/j.ijid.2016.02.001 26854199

[13] Attaran A, Caplan A, Gaffney C, Igel L. Open letter to Dr. Margaret Chan, Director-General, WHO. [cited 20 March 2018]. Available at: www.washingtonpost.com/news/to-your-health/wp-content/uploads/sites/26/2016/05/Zika-Olympics-Open-Letter-to-WHO-current2.pdf

[14] Castro MC. Zika virus and the 2016 Olympic Games—Evidence-based projections derived from dengue do not support cancellation. Travel Med Infect Dis. 2016;14:384–8. 10.1016/j.tmaid.2016.06.007 27363326

[15] Lewnard JA, Gonsalves G, Ko AI. Low Risk of International Zika Virus Spread due to the 2016 Olympics in Brazil. Ann Intern Med. 2016;165:286–7. 10.7326/M16-1628 27454521 PMC5444538

[16] Codeco C, Villela D, Gomes MF, Bastos L, Cruz O, Struchiner C, et al Zika is not a reason for missing the Olympic Games in Rio de Janeiro: response to the open letter of Dr Attaran and colleagues to Dr Margaret Chan, Director—General, WHO, on the Zika threat to the Olympic and Paralympic Games. Mem Inst Oswaldo Cruz. 2016;111:414–5. 10.1590/0074-02760160003 27304097 PMC4909043

[17] World Health Organization. WHO public health advice regarding the Olympics and Zika virus. [cited 20 March 2018]. Available at: www.who.int/mediacentre/news/releases/2016/zika-health-advice-olympics/en/

[18] World Health Organization. Zika situation report (25 August 2016). [cited 20 March 2018]. Available at: http://www.who.int/emergencies/zika-virus/situation-report/25-august-2016/en/

[19] Nakamura S, Wada K, Yanagisawa N, Smith DR. Health risks and precautions for visitors to the Tokyo 2020 Olympic and Paralympic Games. Travel Med Infect Dis. 2018;22:3–7. 10.1016/j.tmaid.2018.01.005 29360525

[20] Kutsuna S, Kato Y, Moi ML, Kotaki A, Ota M, Shinohara K, et al Autochthonous dengue fever, Tokyo, Japan, 2014. Emerg Infect Dis. 2015;21:517–20. 10.3201/eid2103/141662 25695200 PMC4344289

[21] Fukusumi M, Arashiro T, Arima Y, Matsui T, Shimada T, Kinoshita H, et al Dengue Sentinel Traveler Surveillance: Monthly and Yearly Notification Trends among Japanese Travelers, 2006–2014. PLoS Negl Trop Dis. 2016;10:e0004924 10.1371/journal.pntd.0004924 27540724 PMC4991785

[22] Mogi M, Tuno N. Impact of climate change on the distribution of Aedes albopictus (Diptera: Culicidae) in northern Japan: retrospective analyses. J Med Entomol. 2014;51:572–9. 24897849 10.1603/me13178

[23] Japan National Tourist Organization. [cited 20 March 2018]. Available at: https://www.jnto.go.jp/jpn/statistics/marketingdata_tourists_after_vj.pdf

[24] Bhatt S, Gething PW, Brady OJ, Messina JP, Farlow AW, Moyes CL, et al The global distribution and burden of dengue. Nature. 2013;496:504–7. 10.1038/nature12060 23563266 PMC3651993

[25] Stanaway JD, Shepard DS, Undurraga EA, Halasa YA, Coffeng LE, Brady OJ, et al The global burden of dengue: an analysis from the Global Burden of Disease Study 2013. Lancet Infect Dis. 2016;16:712–23. 10.1016/S1473-3099(16)00026-8 26874619 PMC5012511

[26] Centers for Disease Control and Prevention. Dengue virus infections: 2015 Case definition. [cited 20 March 2018]. Available at: https://wwwn.cdc.gov/nndss/conditions/severe-dengue/case-definition/2015

[27] World Health Organization. Dengue and severe dengue fact sheet. [cited 20 March 2018] Available at: http://www.who.int/mediacentre/factsheets/fs117/en/

[28] Castro MC, Wilson ME, Bloom DE. Disease and economic burdens of dengue. Lancet Infect Dis. 2017;17:e70–e8. 10.1016/S1473-3099(16)30545-X 28185869

[29] Duong V, Lambrechts L, Paul RE, Ly S, Lay RS, Long KC, et al Asymptomatic humans transmit dengue virus to mosquitoes. Proc Natl Acad Sci USA. 2015;112:14688–93. 10.1073/pnas.1508114112 26553981 PMC4664300

[30] Society of Automotive Engineers, J1739: Potential Failure mode and Effects Analysis in Design (Design FMEA), Potential Failure Mode and Effects Analysis in Manufacturing and Assembly Processes (Process FMEA) [cited 20 March 2018]. Available at: https://www.sae.org/standards/content/j1739_200901/

[31] US Department of Defence, MIL-STD-1629A: Procedures for Performing a Failure Mode Effects and Criticality Analysis. 1980. [cited 20 March 2018]. Available at: https://src.alionscience.com/pdf/MIL-STD-1629RevA.pdf

[32] Goodrum L, Varkey P. Prevention is better: the case of the underutilized failure mode effect analysis in patient safety. Isr J Health Policy Res. 2017;6:10 10.1186/s13584-016-0131-2 28239449 PMC5319099

[33] Lu Y, Teng F, Zhou J, Wen A, Bi Y. Failure mode and effect analysis in blood transfusion: a proactive tool to reduce risks. Transfusion. 2013;53:3080–7. 10.1111/trf.12174 23560475

[34] Lago P, Bizzarri G, Scalzotto F, Parpaiola A, Amigoni A, Putoto G, et al Use of FMEA analysis to reduce risk of errors in prescribing and administering drugs in paediatric wards: a quality improvement report. BMJ open. 2012;2:e001249 10.1136/bmjopen-2012-001249 23253870 PMC3533113

[35] Ministry of Health, Labour and Welfare. [cited 20 March 2018]. Available at: http://www.mhlw.go.jp/toukei/saikin/hw/iryosd/m17/is1701.html

[36] Ministry of Health, Labour and Welfare. National Action Plan on Antimicrobial Resistance, 2016–2020 [cited 20 March 2018]. Available at: http://www.mhlw.go.jp/file/06-seisakujouhou-10900000-Kenkoukyoku/0000138942.pdf

[37] Ministry of Health, Labour and Welfare. 2016 Survey Summary on Physicians, Dentists, and Pharmacists [cited 20 March 2018]. Available at: http://www.mhlw.go.jp/toukei/list/33-20.thml

[38] Kutsuna S, Yamamoto K, Takeshita N, Hayakawa K, Kato Y, Kanagawa S, et al Experiences of Response Measures against the 4 Suspected Cases of Ebola Virus Disease from West Africa in the National Center for Global Health and Medicine, Tokyo, Japan. Jpn J Infect Dis. 2018;71:62–64. 10.7883/yoken.JJID.2016.508 29093311

[39] National Institute of Infectious Diseases. [cited 20 March 2018]. Available at: https://www.niid.go.jp/niid/images/epi/dengue/Mosquito_Mediated_161220-4-2.pdf

[40] National Institute of Infectious Diseases: Notification trends among imported degue cases is Japan [cited 20 March 2018]. Available at: https://www.niid.go.jp/niid/ja/dengue-m/690-idsc/6663-dengue-imported.html

[41] The Japanese Association for Infectious Diseases. [cited 20 March 20, 2018]. Available at: www.kansensho.or.jp/mosquito/medical_list.html

[42] Japan Medical Service Accreditation for International Patients. [cited 20 March 2018]. Available at: http://jmip.jme.or.jp/index.php?l=eng

[43] Japan Institute for Global Health. [cited 20 March 2018]. Available at: http://jigh.org

[44] Tokyo Metroplitan Government. [cited 20 March 2018]. Available at: http://www.himawari.metro.tokyo.jp/qq13/qqport/tomintop/other/fks230.php

[45] Japan National Tourism Organization. JAPAN: the Official Guide [cited 20 March 2018]. Available at: https://www.jnto.go.jp/eng/basic-info/emergency-info/if-you-become-ill.html

[46] Ministry of Health, Labour and Welfare. Information on Hospitals/Clinics [cited 20 March 2018]. Available at: http://www.mhlw.go.jp/english/policy/health-medical/medical-care/info_hpspitals.html

[47] Ministry of Health, Labour, and Welfare. [cited March 20, 2018]. Available at: www.mhlw.go.jp/stf/seisakunitsuite/bunya/0000141689.html

[48] Ministry of Health, Labour and Welfare. [cited 20 March 2018]. Available at: http://www.mhlw.go.jp/stf/seisakunitsuite/bunya/kenkou_iryou/kenkou/seikatsu-eisei/seikatsu-eisei03/03.html

[49] Ministry of Health, Labour and Welfare. [cited 20 March 2018]. Available at: http://www.mhlw.go.jp/stf/houdou/0000153493.html

[50] Japanese Toursim Agency. [cited 20 March 2018]. Available at: www.mlit.go.jp/kankocho/shisaku/sangyou/juutaku-shukuhaku.html

[51] Ministry of Land, Infrastructure, Transport and Tourism. [cited 20 March 2018]. Available at: http://www.mlit.go.jp/kankocho/minpaku/

[52] Rezza G, Nicoletti L, Angelini R, Romi R, Finarelli AC, Panning M, et al Infection with chikungunya virus in Italy: an outbreak in a temperate region. Lancet. 2007;370:1840–6. 10.1016/S0140-6736(07)61779-6 18061059

[53] Ciota AT, Bialosuknia SM, Zink SD, Brecher M, Ehrbar DJ, Morrissette MN, et al Effects of Zika Virus Strain and Aedes Mosquito Species on Vector Competence. Emerg Infect Dis. 2017;23:1110–7. 10.3201/eid2307.161633 28430564 PMC5512477

[54] Liu Z, Zhou T, Lai Z, Zhang Z, Jia Z, Zhou G, et al Competence of Aedes aegypti, Ae. albopictus, and Culex quinquefasciatus Mosquitoes as Zika Virus Vectors, China. Emerg Infect Dis. 2017;23:1085–91. 10.3201/eid2307.161528 28430562 PMC5512498

[55] Watts N, Amann M, Ayeb-Karlsson S, Belesova K, Bouley T, Boykoff M, et al The Lancet Countdown on health and climate change: from 25 years of inaction to a global transformation for public health. Lancet. 2017 10 30 pii: S0140-6736(17)32464–9. 10.1016/S0140-6736(17)32464-9 29096948

