# Supplementary material for: Health preparedness plan for dengue detection during the 2020 summer Olympic and Paralympic games in Tokyo
Source: PLoS Negl Trop Dis. 2018 Sep 20;12(9):e0006755. doi: 10.1371/journal.pntd.0006755 (PMC6147396; doi:10.1371/journal.pntd.0006755)
Supplement: S1 Table — (DOCX) [file pntd.0006755.s001.docx]

**S1 Table. Criteria for the Evaluation of Severity Component**

| Severity | Characterization | Ranking |
| --- | --- | --- |
| Hazardous without warning | Failure creates a severe disruption to personal health and/or have serious public health impact. The disease occurs without any warnings. There is no cure and/or established disease-specific treatment/prevention for the disease. | 10 |
| Hazardous with warning | Failure creates a severe disruption to personal health and/or have serious public health impact. The disease occurs with warning signs. There is no cure and/or established disease-specific treatment/prevention for the disease. | 9 |
| Very high | Failure creates a severe disruption to personal health and/or have serious public health impact. The disease occurs with warning signs. There is a cure and/or established disease-specific treatment/prevention for the disease. | 8 |
| High | Failure creates a moderate disruption to personal health that lasts for a long period of time (more than one month) and have moderate/serious public health impact. There may or may not be a cure and/or established disease-specific treatment/prevention for the disease. | 7 |
| Moderate | Failure creates a moderate disruption to personal health that lasts for a short period of time (days to weeks) and/or have moderate/severe public health impact. There is no cure and/or established disease-specific treatment/prevention for the disease. | 6 |
| Low | Failure creates a moderate disruption to personal health that lasts for a short period of time (days to weeks) and/or have moderate/severe public health impact. There is a cure and/or established disease-specific treatment/prevention for the disease. | 5 |
| Very Low | Failure creates a moderate disruption to personal health and have minimal public health impact. There may or may not be a cure and/or established disease-specific treatment/prevention for the disease. | 4 |
| Minor | Failure creates a minor disruption to personal health and/or have minimal public health impact. There is no cure and/or established disease-specific treatment/prevention for the disease. | 3 |
| Very minor | Failure creates a minor disruption to personal health and/or have minimal public health impact. There is a cure and/or established disease-specific treatment/prevention for the disease. | 2 |
| None | No health effects. There are no noticeable symptoms. | 1 |
