# Supplementary material for: Health preparedness plan for dengue detection during the 2020 summer Olympic and Paralympic games in Tokyo
Source: PLoS Negl Trop Dis. 2018 Sep 20;12(9):e0006755. doi: 10.1371/journal.pntd.0006755 (PMC6147396; doi:10.1371/journal.pntd.0006755)
Supplement: S2 Table — (DOCX) [file pntd.0006755.s002.docx]

**S2 Table. Criteria for the Determination of Occurrence Component**

| Occurrence | Characterization | Ranking |
| --- | --- | --- |
| Very high with no information | Failure is almost inevitable with no information of any kind. We don’t have any information about the occurrence of a particular problem, but laws of physics or science suggest that it is likely that we have a very high occurrence for this failure mode. | 10 |
| Very high with limited information | Failure is almost inevitable based on some non-objective information or computer models. We don’t have any solid statistics about the occurrence, but there is some empirical information or computer models that indicate that failure is very likely to be high. | 9 |
| Very high with objective information. | Failure is almost inevitable and information has been characterized based on records. We have solid statistics about the high occurrence of a certain problem, the problem is very likely to occur and it is quite predictable if certain conditions are met. | 8 |
| High with no information | Generally associated with processes similar to previous processes which have often failed. Failure occurrence is likely to be high for our process but has not been characterized yet. There is no information of any kind and the likelihood is uncertain and unpredictable. | 7 |
| High with objective information | Generally associated with processes similar to previous processes which have often failed. There is a high likelihood that something might happen based on facts, scientific measurements, computer models or recorded information of some kind. | 6 |
| Moderate with no information | Generally associated with processes similar to previous processes which have experienced occasional failures, but not in major proportions. Failure occurrence is likely to be moderate for our process but has not been characterized yet. There is no information about the likelihood that this problem will occur. | 5 |
| Moderate with objective information | Generally associated with processes similar to previous processes which have experienced occasional failures, but not in major proportions. There is a moderate likelihood that something might happen based on facts, scientific measurements, computer models or recorded information of some kind. | 4 |
| Low with no information | Isolated failures associated with similar processes. Failure occurrence is likely to be low for our process but has not been characterized yet. There is no information about the likelihood that this problem will occur. | 3 |
| Low with objective information | Isolated failures associated with similar processes. There is a low likelihood that something might happen based on facts, scientific measurements, computer models or recorded information of some kind. | 2 |
| Remote | Failure unlikely. No failures ever associated with almost identical processes. | 1 |
