# Supplementary material for: Health preparedness plan for dengue detection during the 2020 summer Olympic and Paralympic games in Tokyo
Source: PLoS Negl Trop Dis. 2018 Sep 20;12(9):e0006755. doi: 10.1371/journal.pntd.0006755 (PMC6147396; doi:10.1371/journal.pntd.0006755)
Supplement: S3 Table — (DOCX) [file pntd.0006755.s003.docx]

**S3 Table. Criteria for the Determination of Detection Component**

| Detection | Likelihood that the existence of a failure mode will be detected by process controls, detection systems before next or subsequent process, or before it causes a human health problem | Ranking |
| --- | --- | --- |
| Almost impossible | No known control(s) available to detect failure mode (nothing in place to detect a potential problem) | 10 |
| Very remote | Very remote likelihood that current controls will detect failure mode (current controls are for other failure modes, but might detect this failure sometimes) | 9 |
| Remote | Remote likelihood that current controls will detect failure mode (current controls are for this failure mode, but are highly restricted, intermittent or insufficient) | 8 |
| Very low | Very low likelihood that current controls will detect failure mode (current controls are for this failure mode, but are restricted, intermittent or insufficient such as continuous visual inspection) | 7 |
| Low | Low likelihood that current controls will detect failure mode (current controls are set for this failure mode, but have very low accuracy) | 6 |
| Moderate | Moderate likelihood that current controls will detect failure mode (current controls for this failure mode are very simple and not very effective) | 5 |
| Moderately high | Moderate likelihood that current control(s) will detect failure mode. | 4 |
| High | High likelihood that current control(s) will detect the failure mode. | 3 |
| Very high | Very high likelihood that current control(s) will detect the failure mode. | 2 |
| Almost certain | Current control(s) almost certain to detect failure mode. Reliable detection controls are known in similar processes or activities. | 1 |
